# Quantification of Outcrossing Events in Haploid Fungi Using Microsatellite Markers

**DOI:** 10.3390/jof6020048

**Published:** 2020-04-14

**Authors:** Dong-Hyeon Lee, Brenda D. Wingfield, Jolanda Roux, Michael J. Wingfield

**Affiliations:** 1 Department of Biochemistry, Genetics, and Microbiology, Forestry and Agricultural Biotechnology Institute (FABI), University of Pretoria, Pretoria 0028, South Africa; Mike.wingfield@fabi.up.ac.za; 2 Division of Forest Diseases and Insect Pests, National Institute of Forest Science, Seoul 02455, Korea; 3 Department of Plant and Soil Sciences, FABI, University of Pretoria, Pretoria 0028, South Africa; jolanda.roux@sappi.com

**Keywords:** genotypic diversity, population, reproduction, sex

## Abstract

Species in genera of the fungal family Ceratocystidaceae are known to have different mating strategies, including heterothallism and homothallism. Of these, species of *Ceratocystis*, typified by the pathogen *Ceratocystis fimbriata* all undergo unidirectional mating-type switching. This implies that the pathogens possess the ability to self, but also to undergo sexual outcrossing between isolates of different mating types. In this study, we extended the recently developed microsatellite-based technique to determine the extent to which outcrossing occurs in ascospore masses of haploid fungi to two field collections of *Ceratocystis albifundus*. In this way, the role of reproductive strategies in shaping population structure and diversity could be better understood. Results showed that a high frequency of outcrossing occurs in isolates of the pathogen from both non-native and native areas. This explains the high level of genetic diversity previously observed in this population despite the fact that this pathogen has the ability to self.

## 1. Introduction

A broad range of reproductive strategies are found in filamentous ascomycete fungi [1,2,3]. This results in a wide variety of life cycles and a high level of reproductive plasticity emerging from either sexual or asexual propagation [4]. In addition to sexual reproduction that ensures some levels of genetic exchange [5], asexual reproduction via mycelium or mitospores (conidia) is widespread in fungi [3].

Despite the time investment needed to locate a mating partner [2] and the energy invested to produce sexual structures [6], the question as to why sexual reproduction has been retained in some species is one of the long-standing conundrums concerning fungal mating systems. In this regard, *Ceratocystis* species are particularly interesting because they all undergo unidirectional mating-type switching. Consequently, they have the ability to either self or undergo sexual outcrossing [7,8,9]. Although sexual reproduction could be a disadvantage due to its relative cost, *Ceratocystis* spp. are able to fully benefit from sexual recombination, producing novel recombinant genotypes that enable them to successfully exploit diverse ecological environments [8,10,11,12,13].

*Ceratocystis albifundus* is an important fungal pathogen, best known as the cause of a serious stem canker and wilt disease of *Acacia mearnsii* (black wattle) in southern and eastern Africa [12,14]. It also causes a serious canker disease in *Protea cynaroides* farmed for cut-flowers in the region [15]. In order to determine the extent to which outcrossing occurs either in vitro or in nature, a microsatellite-based technique has recently been developed for *C. albifundus* by Lee et al. [9]. 

Although it has been shown previously that 27% outcrossing (4 out of 15 isolates) occurs in *C. albifundus* under natural conditions [9], the extent of this outcrossing is not known. The aim of this study was thus to consider the role of reproduction in shaping the population structure in the pathogen. The extent to which outcrossing occurs was determined using new collections of *C. albifundus* from native trees in the Kruger National Park of South Africa and a plantation of non-native *A. mearnsii* trees.

## 2. Materials and Methods 

Isolates for this study included those from non-native *A. mearnsii* trees in a plantation in the Bloemendal area (KZN) close to Pietermaritzburg, South Africa (RSA). In addition, isolates were collected from native trees growing naturally at three locations (Pretoriuskop, Lower Sabie and Tsohowane) in the Kruger National Park (KNP), as shown in Table 1. 

More than fifty single ascospore mass isolations were made from a total of 18 trees following the technique described by Lee et al. [12]. This resulted in a total of 205 ascospore mass cultures of *C. albifundus* as shown in Table 1. In the plantation situation, isolations were made from the stumps of ten recently felled *A. mearnsii* trees, which had been grown approximately 5 m apart. In the KNP where the trees occurred naturally, the isolations made from eight randomly selected trees (in each of the three areas) where ascomata of *C. albifundus* were visible on freshly exposed wounds. All the cultures used in this study were deposited in the culture collection (CMW) of the Forestry and Agricultural Biotechnology Institute, University of Pretoria, RSA, as displayed in Table 1.

To ensure the correct identity of all isolates obtained, cultures were subjected to identification using both morphological characters and internal transcribed spacer (ITS) barcoding sequences as described previously [12,16]. Isolates were initially recognized as those of *C. albifundus* based on their characteristic light-colored ascomatal bases, bearing black necks and producing hat-shaped ascospores [16]. The morphologically identified isolates from which single ascospore cultures were made were then sequenced, and these sequences were used in a BLASTn analysis against the nucleotide database of the National Center for Biotechnology Information (NCBI) (http://blast.st-va.ncbi.nlm.nih.gov/Blast.cgi) as described by Lee et al. [12]. Genomic DNA was extracted from all the single ascospore cultures obtained in this study, following the cetyltrimethylammonium bromide (CTAB) based-protocol described by Möller et al. [17]. Polymerase chain reactions (PCR) for species identification were as described by Lee et al. [9,12].

To quantify the frequency of outcrossing events in the populations of *C. albifundus*, genescan analyses were carried out as described by Lee et al. [9]. Briefly, single ascospore masses taken from the apices of *C. albifundus* ascomata were used for DNA extraction with 10 % Chelex 100 (Bio Rad Laboratories, Hercules, CA, USA) as described by Walsh et al. [18]. Microsatellite amplifications were then performed using two primer sets, AG 7/8 and AG 15/16, as shown in Table 2, to determine whether either a haploid microsatellite profile with two different single alleles or a profile with more than one allele per microsatellite locus could be observed. This would then indicate whether the ascospore masses were the result of a self or from an outcrossing event between two genetically different individuals of *C. albifundus*.

## 3. Results and Discussion

*Ceratocystis albifundus* was relatively common on the freshly cut stumps of non-native *A. mearnsii* trees, while the pathogen was found less frequently on wounds of native trees in the KNP. A total of 205 cultures of *C. albifundus* were recovered, of which 116 and 89 ascospore mass cultures were from KZN and KNP, respectively. All cultures produced sexual structures on malt extract agar (MEA) in Petri dishes, which had the typical morphological features of the fungus. A BLAST search using ITS sequence data confirmed the identity of selected isolates as *C. albifundus*. The sequence data obtained from three randomly selected single ascospore cultures were deposited in NCBI (Accession no. MH685552‒685554). 

**Figure 1:**
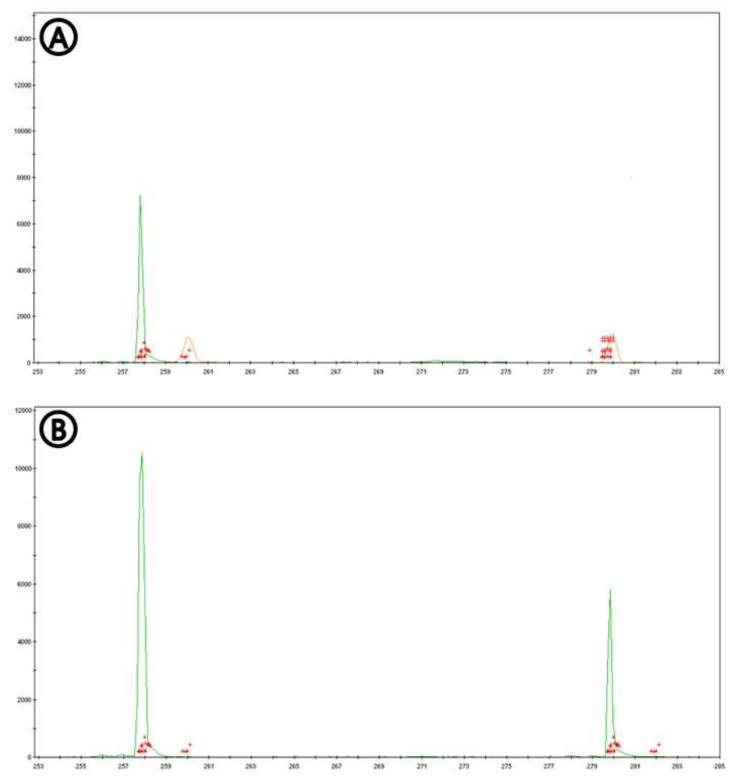
The results of allele scoring obtained from the primer labelled with a fluorescent dye, VIC (green) in GeneMarker ver.2.2.0 (SoftGenetics, State College, PA, USA); GeneScan™ 600 LIZ (Applied Biosystem, Foster City, CA, USA) was used as the internal size standard (orange); some artefactual peaks are indicated as the red crosses. (A): microsatellite profile with no evidence of outcrossing (a single allele; haploid microsatellite profile), (B): microsatellite profile with evidence of outcrossing (two different sizes of alleles; diploid microsatellite profile).

The four microsatellite primers shown in Table 2 resulted in the expected amplicon size shown in Figure 1. In cases where isolates were obtained from the non-native *A. mearnsii* in KZN, the microsatellite primer pair AG7/8 generated two different allele sizes (258 bp and 280 bp, respectively). A total of 50 cultures had this microsatellite profile, indicating that an outcrossing event had occurred (43.1% of outcrossing frequency). For the microsatellite primer pair AG15/16, three different allele sizes (272 bp, 286 bp and 292 bp, respectively) were obtained from these 50 cultures (43.1% of outcrossing frequency). 

In the case of the cultures obtained from KNP, outcrossing was relatively common when the cultures originated from *Terminalia sericea* and *Lannea stuhlmannii*. For the microsatellite primer set AG7/8, two different allele sizes (286 bp and 292 bp, respectively) were observed and four isolates had the microsatellite profile, indicative of outcrossing events having occurred (4% of outcrossing frequency). The microsatellite primer set AG 15/16 produced two different allele sizes (286 bp and 292 bp, respectively), suggesting that 22 isolates had undergone outcrossing (25% outcrossing frequency). Overall, two primer pairs (AG 7/8 and AG 15/16) from each of the study areas consistently generated microsatellite profiles having the two different allele sizes at these loci, as shown in Table 3.

The results of this study confirmed that outcrossing occurs in *C. albifundus* under field conditions. Consequently, that sexual reproduction is common in populations of the pathogen in South Africa, and this is true both for infections on plantation-grown non-native as well as naturally growing native trees. 

An expectation of this study was that outcrossing events would have been more common in the natural KNP population of *C. albifundus* than in artificially planted and non-native *A. mearnsii* trees. As only four microsatellite regions were used in this study, the results are an underestimate of the actual amount of outcrossing and the differences between the native and non-native isolates may in fact not be significant [9]. Nonetheless, the results of this study show that extensive outcrossing occurs in both cases, and surprisingly, that it is somewhat more common in the non-native situation. This is an interesting result given the fact that in culture, the fungus commonly acts homothallically with a large percentage of single ascospores giving rise to fertile cultures. However, this result is not unusual for other homothallic fungi, which have been shown to undergo sexual reproduction in nature.

## Figures and Tables

**Table 1:** Isolates of *Ceratocystis albifundus* used in this study.

Sampling Sites		Host	Isolate Number
Non- native area (*Acacia* planation)	Bloemendal	*Acacia mearnsii*	^a^CMW38486-38527, CMW43527-43595, CMW44082, CMW44111-44115
Native areas (Kruger National Park)	Pretoriuskop	*Terminalia sericea*	CMW 41508-41530, CMW42118-42126, CMW43680
	Tsohowane		CMW 41531-41545, CMW41580-41588,
	Lower Sabie		CMW 41546-41549
	Lower Sabie	*Lannea stuhlmannii*	CMW 41550-41564, CMW 41566-41572, CMW 41574-41579

^a^ Culture collection (CMW) of the Forestry and Agricultural Biotechnology Institute, University of Pretoria

**Table 2:** Microsatellite primers used in this study.

Primers	Sequences	Reference
AG7F ^a^	CGA GAC AGC AAC ACA AGC CC	Barnes et al. [19]
AG8R ^b^	GGG GCG GTG GTG CAA TTG TC	
AG15F	CTT GAC CGA CCT GCC GAT TG	
AG16R	GGA TAG CAG CGA CAA GGA CC	

^a & b^ Forward and reverse, respectively.

**Table 3:** The estimated outcrossing frequency of *Ceratocystis albifundus* in Bloemendal area (KZN) and Kruger National Park (KNP).

Sampling Sites		ID of Bark Flaps	Number of Isolates Successfully Recovered	Total Number of Isolates Showing a Heterozygous Profile	
				AG 7/8	AG 15/16
Non-native area (*Acacia* planation)	Bloemendal	*Acacia mearnsii* 1	7	3	5
		*Acacia mearnsii* 2	11	6	5
		*Acacia mearnsii* 3	12	5	2
		*Acacia mearnsii* 4	14	5	7
		*Acacia mearnsii* 5	9	4	3
		*Acacia mearnsii* 6	13	2	4
		*Acacia mearnsii* 7	15	6	7
		*Acacia mearnsii* 8	13	2	7
		*Acacia mearnsii* 9	12	8	6
		*Acacia mearnsii* 10	10	9	4
	Total		116	50	50
	Outcrossing events observed (%)			43.1%	43.1%
Native areas (Kruger National Park)	Pretoriuskop	*Terminalia sericea* 17-1	7	1	4
		*Terminalia sericea* 17-2	6	1	2
		*Terminalia sericea* 17-9	14	-	6
		*Terminalia sericea* 7-9	6	-	1
	Lower Sabie	*Terminalia sericea* 23	4	-	-
		*Lannea stuhlmannii* 26	28	2	6
	Tsohowane	*Terminalia sericea* 37	15	-	3
		*Terminalia sericea* 38	9	-	-
	Total		89	4	22
	Outcrossing events observed (%)			4.5%	24.7%
